# Orthologous endogenous retroviruses exhibit directional selection since the chimp-human split

**DOI:** 10.1186/s12977-015-0172-6

**Published:** 2015-06-20

**Authors:** Patrick Gemmell, Jotun Hein, Aris Katzourakis

**Affiliations:** Department of Zoology, University of Oxford, Oxford, UK; Department of Statistics, University of Oxford, Oxford, UK

**Keywords:** Endogenous retrovirus (ERV), Selection, HERV-H

## Abstract

**Background:**

Endogenous retroviruses (ERVs) are often viewed as selfish DNA that do not contribute to host phenotype. Yet ERVs have also been co-opted to play important roles in the maintenance of stem cell identity and placentation, amongst other things. This has led to debate over whether the typical ERV confers a cost or benefit upon the host. We studied the divergence of orthologous ERVs since the chimp-human split with the aim of assessing whether ERVs exert detectable fitness effects.

**Results:**

ERVs have evolved faster than other selfish DNA in human and chimpanzee. The divergence of ERVs relative to neighbouring selfish DNA is positively correlated with the length of the long terminal repeat of an ERV and with the percentage of neighbouring DNA that is not selfish. ERVs from the HERV-H family have diverged particularly quickly and in a manner that correlates with their level of transcription in human stem cells. A substitution into a highly transcribed HERV-H has a selective coefficient of the order of 10^−4^. This is large enough to suggest these substitutions are not dominated by drift.

**Conclusions:**

ERVs differ from other selfish DNA in the extent to which they diverge and appear to have measurable effects on hosts, even after fixation. The effects are strongest for HERV-H and suggest that the HERV-H transcriptome has recently evolved under the influence of directional selection. As there are many HERV-H loci distributed across the ape lineage, our results suggest that in future this family can be used to model the evolutionary consequences of ERV exaptation in primates and other mammals.

**Electronic supplementary material:**

The online version of this article (doi:10.1186/s12977-015-0172-6) contains supplementary material, which is available to authorized users.

## Background

As an obligate part of their lifecycle, retroviruses integrate their genomes into their host’s nuclear DNA. This integrated retroviral genome is referred to as a provirus. Sometimes integration occurs in a germ line cell, and if the integration is not too damaging to the host, then it becomes possible for proviral DNA to be passed in a vertical (Mendelian) way from parent to offspring. An initial vertical transmission is known as an endogenization and the inherited proviral DNA is known as an endogenous retrovirus (ERV). Over time, some ERVs reach high frequencies or fixation in a host population and it is therefore possible to detect the traces of ancient viral infections, often in fragmented form, by examining modern genomes.

As transposable elements (TEs) with an RNA intermediate form, ERVs can be thought of as selfish DNA [1, 2]. The term ‘selfish DNA’ refers to sequences that are present in genomes in multiple copies largely due to their ability to replicate themselves rather than because they provide any benefit to the host. Although selfish entities can replicate, they do not seem to expand genomes indefinitely, probably because they impose a selective cost [3, 4]. Selection will act against individual TEs, especially if they are very harmful. This selective cost has lead to the evolution of host defences [5]. Host defences are not perfect however, and TEs can still saturate a genome unless selection against them increases sufficiently quickly with respect to mean element copy number per individual [6, 7]. In other words, as TEs do not fill up our genomes, population genetics suggests they must be harmful, and as TEs can increase their copy number, some fraction of TEs may fix, even when they have a cost to their host.

The cost of harbouring TEs is often categorized as arising in three ways [8], all of which apply to ERVs. The first cost of TEs is due to the fact that they can be present in many copies in the genome. As repetitive sequence they may increase the occurrence of ectopic recombination whereby meiotic crossover occurs between TEs from the same family that are located in non-homologous parts of the genome [9]. The probability of ectopic recombination between two sequences is thought to be related to length of uninterrupted similarity between them [10], and as ERVs are longer than typical TEs, two particular ERVs may be more likely to ectopically recombine than, say, two particular SINEs. The second cost of TEs is due to the possibility that an element may insert itself into a functional region of the genome in a way that disrupts the ability of the host to survive. Insofar as ERVs retain their ability to retrotranspose (i.e. insert a copy of themselves into a new chromosomal location within a cell) or to reinfect (i.e. insert a copy of themselves in a potentially different cell after performing a cell exit and subsequent cell entry), it is clear that ERVs present the same risks as other TEs in this respect. The third cost of TEs is the cost to the host due to the mechanism of replication itself. For ERVs, particularly recently integrated ones, this cost may be severe, as ERVs contain viral genes that were selected to allow exogenous viruses to circulate between hosts. This means that in addition to the side-effects that are common to all retrotransposons, such as those due to the production of an intermediate RNA form, ERVs can have additional effects. An example of an additional effect is virion formation, the costs of which can include immune responses or the infection and mutagenization of cells throughout the body [11]. Indeed, it is ERVs that mitigate the consequences of their history as horizontally infectious agents by losing their envelope gene that are exactly those that proliferate most effectively in the long term [12].

Despite the ways in which ERVs can be harmful, there are an increasing number of described cases where ERVs may be conferring some benefits to their host. For example, recent debate has occurred over the significance of the fact that ERVs exhibit relatively high levels of placental transcription [13–17], the fact that some retroviral promoters are exclusively expressed in the placenta [18, 19], and the fact that genes derived from ERVs have frequently been co-opted for placental function [20]. One suggestion, as proposed by [21], is that ERVs and the placenta are in symbiosis: placental expression of ERVs is tolerated because ERVs were involved in the origin of the placenta via the creation of the trophoblast cell lineage and because, since then, ERVs have continued to play important roles in placental function. It is argued that long terminal repeats (LTRs) of ERVs act as mobile promoters that can rapidly rewire gene regulation networks in a way that may be crucial to the origin and evolution of a new cell type. This hypothesis is interesting but controversial [22, 23] as from a viral perspective placental expression may allow ERVs to segregate with greater than even odds from heterozygous mothers and also provide a mechanism by which a father can infect a mother and all of her future offspring.

A more concrete example of exaptation also hinges on the ability of ERVs to facilitate widespread transcriptional rewiring and comes from studies that highlight the participation of ERVs in the initiation and maintenance of stem cell identity. It has been shown that of 1225 full-length copies of HERV-H in the human genome, 550 are actively transcribed in human pluripotent stem cells at levels that are positively correlated with the integrity of their 5′ LTRs [24]. In human embryonic stem cells, the transcription factor LBP9 has been shown to drive production of stem cell specific HERV-H associated chimeric transcripts and long non-coding RNAs (lncRNAs), the latter having been shown to be essential for the maintenance of a stem cell like state [24]. Elsewhere it has been independently shown that HERV-H knockdown downregulates pluripotency markers, and that HERV-H transcription is necessary for both the creation and maintenance of stem cell identity [25]. Furthermore, a large scale analysis of both the mouse and the human stem cell transcriptome suggests that LTR derived transcripts are under the direct control of the main stem cell specific transcription factors [26]. Research on mouse has produced related results, and the MuERV-L family of ERVs has been shown to produce chimeric transcripts originating from over 300 LTR loci, the activity of which appear to grant some totipotent like properties to induced and embryonic stem cells [27]. The weight of evidence from these studies does suggest that, at least for some part of their history, a proportion of ERVs have contributed in important ways to host function.

In this paper, we consider the degree to which ERVs in general are active parts of the genome rather than inert sequences that lost their effects on hosts prior to fixation. Given viruses and TEs can be so disruptive to the host, ERVs that are observed in contemporary genomes have often been assumed to be effectively harmless and to evolve neutrally. However, we do not have a clear picture of the costs, benefits and frequency of ERVs in ancient populations that are necessary to support such assumptions. At one extreme, some ERVs we observe today may be members of families that were both prolific and harmful in ancestral populations, so that the fixation of some deleterious ERVs was an inevitable consequence of their ability to replicate quickly. On the other hand, ERVs may have been frequently co-opted due to the pre-packaged functions they provided, with the benefits of these functions balancing out any deleterious side effects. In this study we examine orthologous ERVs in human and chimpanzee genomes and compare their divergence since the split between the two species. If ERVs are indeed inert they should have evolved neutrally after they reached fixation. On the other hand, if ERVs had an effect on the host they should have evolved at rates that differ from the neutral rate. In particular, ERVs that are conserved will have evolved more slowly than the neutral rate while ERVs should only have evolved more quickly than expected if they were useful to the host and underwent adaptation, or if they were still harmful to the host and were degraded.

## Results

We wanted to see if recently integrated proviruses accumulated mutations more quickly than neighbouring DNA. Our approach was to examine substitutions into ERVs and their neighbouring genomic sequence that lead to differences between human and chimpanzee. To achieve this goal we identified ERVs and their flanking DNA from both species. Using bioinformatics tools, we searched the human and chimpanzee genomes for full-length ERVs using a broad spectrum of retroviral probes. We then attempted to associate the results of our search process in terms of orthology: by using a two stage pairwise alignment process we deemed sufficiently similar sequences originating from syntenic chromosomes in different species as paired orthologues. In the rare case that there was evidence of paralogy we excluded all the paralogous regions from the study. Overall, we identified 336 chimp-human pairs of sequence from a variety of genomic locations (Table 1). The ERVs in the sequence were from a variety of families (Table 2). We carefully pairwise aligned these ERV containing sequences, masking regions that were badly aligned and could not be safely included in the study.

**Table 1:** Chimp-human orthologue linkage. We detected 336 pairs of ERV containing sequence from chimpanzee and human genomes

Linkage	Count
1	29
2	29
3	38
4	22
5	11
6	25
7	36
8	22
9	9
10	11
11	16
12	10
13	8
14	10
15	7
16	5
17	2
18	2
19	19
20	2
21	8
22	0
X	15

Note: ch2a/2b (chimpanzee) were paired with ch2 (human)

**Table 2:** Chimp-human orthologue family, by linkage. ERV family was assigned using the best matching viral *pol* probe (see Detecting ERVs in Methods)

Family	Autosomal	X-linked
ERV-9	57	1
HERV-ADP	1	0
HERV-E	10	1
HERV-F type_b	2	0
HERV-H	58	6
HERV-I	23	0
HERV-K(HML2)	101	4
HERV-K(HML5)	11	1
HERV-K(HML6)	12	0
HERV-K(HML9)	1	0
HERV-L	1	0
HERV-P	2	0
HERV-R	5	0
HERV-T	4	0
HERV-U3	1	0
HERV-W	19	0
HERV-XA	1	0
RRHERV-I	6	1
Unclassified	6	1

Each of the 336 pairs of ERVs in our study are contained in a 40 kb region of DNA. Inspection of these regions reveals they are mostly comprised of repetitive elements. Some of these repetitive elements are typically selfish (e.g. DNA transposons) whereas a minority (e.g. tRNA) are essential to the host. Substitution into regions that are useful to the host will generally be constrained as mutations in these regions are likely to be deleterious. We are interested in whether substitutions into ERVs are more common than substitutions into other selfish elements. To determine this we classified all columns of our alignments as one of: provirus (PV); repetitive and selfish DNA (RM+); and non-repetitive or repetitive but non-selfish (RM-). The sequence classified as PV was the result of our original search for ERVs and the categories RM+ and RM- were assigned to the flanking regions of ERVs by using RepeatMasker annotations. Because CpG sites are known to mutate quickly, we censored these sites in our analyses; all results pertain to censored analyses unless we explicitly state otherwise. Overall, the following site patterns were observed for each of the three categories of sequence (Table 3 and Additional file 1: Table S1).

**Table 3:** Site patterns observed across CpG censored alignments. Patterns were observed at sites classified as one of: ERV (PV); selfish DNA (RM+); or non-repetitive or repetitive but non-selfish (RM-)

Chimp:human	Autosomal			X-linked		
	PV	RM+	RM-	PV	RM+	RM-
A:A	591392	1603551	1145366	26509	83350	43123
A:T	888	2496	1390	38	105	45
A:G	3650	8749	5067	112	335	155
A:C	1104	2678	1593	41	111	46
A:?	0	0	0	0	0	0
T:A	984	2572	1456	28	90	41
T:T	597941	1609580	1151417	26035	87639	43695
T:G	1197	2816	1527	57	109	62
T:C	3636	8534	4949	127	338	172
T:?	0	0	0	0	0	0
G:A	3294	8415	4950	131	389	144
G:T	1143	3034	1688	35	108	51
G:G	450769	1135140	703983	22571	56854	24790
G:C	974	2586	1439	29	105	51
G:?	0	0	0	0	0	0
C:A	1206	2935	1652	26	102	46
C:T	3394	8432	4957	120	328	183
C:G	986	2623	1400	31	91	55
C:C	460550	1128491	698783	18236	56537	25202
C:?	0	0	0	0	0	0
?:A	5	0	2	0	0	0
?:T	0	1	2	0	1	0
?:G	1	0	1	0	1	0
?:C	1	4	0	0	0	0
?:?	0	0	0	0	0	0
total	2123115	5532637	3731622	94126	286593	137861

Hoping to take account of any differences in local mutation rates in the genome, we first considered each of the 336 pairs of virus-containing sequences individually i.e. due to their physical co-location, we considered PV and RM+ as paired measurements. We found that PV divergence is significantly greater than RM+ divergence for autosomal ERVs (Wilcoxon signed-rank test, W = 32602.5, p < 0.0001) with a small median difference of 0.001 substitutions per site. We also found that median PV divergence was greater for autosomal ERVs than for X-linked ERVs (Wilcoxon signed-rank test, W = 3178, p = 0.018) by a distance of 0.002 substitutions per site.

**Fig. 1:**
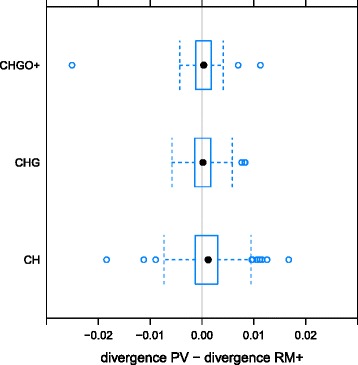
Difference in divergence between PV (ERVs) and RM+ (selfish DNA) for chimp-human orthologues, aggregated by age. Age categories were assigned to chimp-human orthologues by identifying the most distant primate relative in which the orthologous sequence could also be found. Category CH contains ERVs detected in chimpanzee and human only (187 ERVs); category CHG contains ERVs for which gorilla was the most distant relative in which an ERV was detected (77 ERVs); category CHGO+ contains ERVs for which orangutan (70 ERVs) or macaque (2 ERVs) was the most distant relative in which an ERV was detected. (Note: whiskers estimate 95 % confidence intervals, filled dots represent median values, unfilled dots represent outliers.)

As we found that proviruses diverged faster than other selfish DNA we wanted to see if this effect was related to the age of viral integration. To do this we searched for each ERV’s full-length representative in the gorilla, orangutan, and macaque genomes, using the same method as that for the human and chimpanzee. For each chimp-human orthologue we aimed to identify the lineage that split earliest from the lineage leading to chimp/human that also contained the particular ERV in question. In other words, we identified a minimum age bound for each ERV by examining progressively more distant relatives. As this approach relied on the ability of LTR detection software to detect a full-length ERV in more than one species, the age classification was approximate. There were 187 ERVs for which no additional orthologue was found (CH) and 149 ERVs that were confirmed to be at least as old as the gorilla split (CH+): there were 77 ERVs for which gorilla was the earliest split (CHG), 70 ERVs for which orangutan was the earliest split and 2 ERVs for which macaque was the earliest split (CHGO+). Considering the difference between PV divergence and RM+ divergence we found that the potentially youngest ERVs (CH) had diverged significantly more since the chimp-human split than those that were confirmed to be at least as old as the gorilla split (CHG) but that there was no significant difference between PV divergence and RM+ divergence for the CHG and CHGO+ categories (Fig. 1). We therefore report that the potentially youngest ERVs (CH) had diverged significantly more (Wilcoxon signed-rank test, W = 16110, p < 0.01) since the chimp-human split than those that were confirmed to be at least as old as the gorilla split (CH+). The median difference in divergence between PV and RM+ was 0.0012 substitutions per site for alignments in CH and 0.0003 substitutions per site for alignments in CH+.

**Fig. 2:**
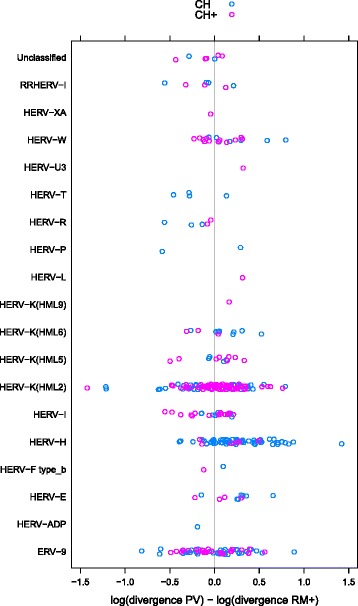
Relative divergence of PV (ERVs) and RM+ (selfish DNA) aggregated by age and ERV family. ERV family was assigned using the best matching viral *pol* probe (see Detecting ERVs in Methods). Age categories were assigned to chimp-human orthologues by identifying the most distant primate relative in which the orthologous sequence could also be found. Category CH contains ERVs detected in chimpanzee and human only (187 ERVs); category CH+ contains ERVs detected in a primate beyond human and chimpanzee (149 ERVs)

As can be seen in (Fig. 2), it appears as if HERV-H is responsible for much of the divergence in the CH category. This was confirmed by re-running our analyses with the 64 HERV-H removed from our dataset. In this case, a significant age effect was no longer observed. Further investigation showed that the difference in divergence between PV and RM+ is significantly greater for HERV-H than for ERVs that are not classified as HERV-H (Wilcoxon signed-rank test, W = 12675, p < 0.0001) with a median difference between PV and RM+ of 0.0026 substitutions per site for HERV-H and 0.0003 substitutions per site for ERVs that are members of any other family. Assuming that substitutions into RM+ are the result of neutral semi-dominant mutations, the ratio of these divergence values suggests a median selection coefficient of 2.3 × 10^−5^ for younger CH ERVs. Moreover, the upper quartile (16 out of 64) of all HERV-H selection coefficients are not small (2*Ns* > 1), ranging from 5 × 10^−5^ to 2 × 10^−4^.

**Fig. 3:**
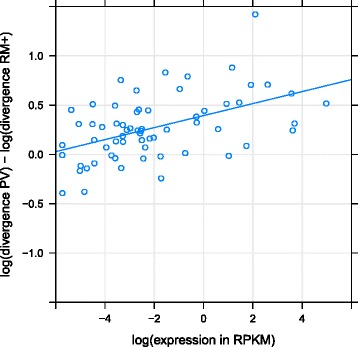
Relative divergence of HERV-H loci and RM+ (selfish DNA) correlates with stem cell transcription. The log of the average transcription level (in reads per kilobase of transcript per million reads mapped) [24] of 63 HERV-H loci across hESC and hiPSC is correlated (R^2^ = 0.23, p < 0.0001) with their divergence since the chimp-human split

**Fig. 4:**
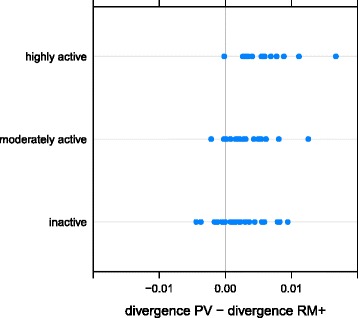
Excess divergence of HERV-H grouped by categorical transcription levels in human stem cells. The difference in divergence between PV (ERVs) and RM+ (selfish DNA) of 63 HERV-H loci (12 “highly active”, 22 “moderately active”, 29 “inactive”) increases with their categorical transcription levels [24] across hESC and hiPSC

The differences we observed between PV and RM+ were quite large. For this reason we examined how divergence related to transcription, for HERV-H orthologues only, and to virus length, LTR length and the percentage of an ERV’s environment that was selfish (RM+) for all orthologues. Pairing our orthologues with transcription activity data [24] we found that the log ratio of PV divergence to RM+ divergence was significantly correlated with the log of the average transcription level of HERV-H in human embryonic stem cells (hESC) and induced pluripotent stem cells (hiPSC) using both linear models (R^2^ = 0.23, p < 0.0001) (Fig. 3) and nonparametric tests (Kendall’s rank correlation, tau = 0.315, p < 0.001). Using the transcription activity categories of [24], we further found that this divergence ratio was higher for 12 “highly-active” ERVs than for 22 “moderately active” ERVs (Wilcoxon signed-rank test, W = 197, p < 0.01), the 22 “moderately active” ERVs in turn had a higher divergence ratio than the 29 “inactive” ERVs (Wilcoxon signed-rank test, W = 424, p = 0.023) (Fig. 4). The median selection coefficients for transcriptionally “highly active”, “moderately active” and “inactive” HERV-H ERVs are 5.7 × 10^−5^, 2.6 × 10^−5^ and 1.3 × 10^−5^ respectively. We further found that, across all ERVs, the log ratio of PV divergence to RM+ divergence was significantly positively correlated with LTR length (Kendall’s rank correlation, tau = 0.121, p < 0.001) and significantly positively correlated with the percentage of the flanking DNA of an ERV that is non-selfish (RM-) (Kendall’s rank correlation, tau = 0.140, p < 0.0001). These correlations remained significant (p < 0.01) even if HERV-H were excluded from our dataset. We did not find a positive correlation between virus length and divergence (Kendall’s rank correlation, tau = −0.10, p = 1.00).

Our results show that ERVs (PV) experience faster evolution than nearby selfish DNA (RM+), particularly if the ERVs are potentially younger (CH), and particularly if they are HERV-H. Our results also show that ERVs evolve faster if they have longer LTRs and are located regions of the genome with less selfish DNA, and that autosomal ERVs evolve faster than X-linked ERVs. The faster evolution of ERVs than nearby selfish DNA might be due to selective forces or to mechanistic factors.

**Fig. 5:**
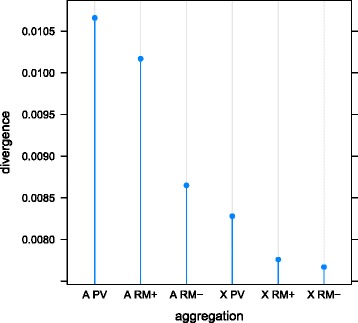
Divergence aggregated by linkage and sequence classification. ERVs (PV) diverge faster than selfish DNA (RM+) which diverges faster than non-repetitive or repetitive but non-selfish DNA (RM-). Autosomal loci (A) diverge faster than X-linked (X) loci

To investigate sex-effects and dominance, as well as the aforementioned mechanistic factors, we aggregated the sequence from our 336 orthologous stretches of ERV containing DNA, combining sequence based on its linkage (autosomal or X-linked) and its classification (PV, RM+ or RM-). We found that ERVs (PV) diverged more quickly than repetitive and selfish flank (RM+), that in turn diverged more quickly than non-repetitive or repetitive but not selfish flanking DNA (RM-) (Table 4, Fig. 5). This was true for the autosome and the X-chromosome, whether or not we censored CpG sites. The divergence values in (Table 4) imply selection coefficients of 1.3 × 10^−5^ and 2.4 × 10^−5^ for autosomal and X-linked ERVs before the censoring of CpG sites and 4.7 × 10^−6^ and 6.7 × 10^−6^ after censoring. We observe that in all cases these are small forces (2*Ns* < 1) and that for both censored and uncensored sites the ratio of autosomal to X-linked relative divergence suggests that mutations into ERVs are recessive.

**Table 4:** Divergence aggregated by class, linkage, CpG censoring, and differences used. Differences used were classified as: all differences (EQ+/−); CG equilibrating differences only (EQ+); and non CG equilibrating differences only (EQ-). Sites were classified as one of: ERV (PV); selfish DNA (RM+); or non-repetitive or repetitive but non-selfish (RM-)

		Uncensored (CpG+)			Censored (CpG-)		
Linkage	Class	EQ+/−	EQ+	EQ-	EQ+/−	EQ+	EQ-
A	PV	0.01649	0.01430	0.00222	0.01066	0.00885	0.00182
A	RM+	0.01446	0.01230	0.00217	0.01017	0.00831	0.00187
A	RM-	0.01144	0.00969	0.00177	0.00865	0.00712	0.00154
X	PV	0.01365	0.01182	0.00184	0.00829	0.00694	0.00135
X	RM+	0.01087	0.00929	0.00159	0.00776	0.00639	0.00137
X	RM-	0.01005	0.00841	0.00166	0.00767	0.00627	0.00140

In our study we make comparisons between ERVs (PV) and repetitive and selfish DNA (RM+) that are paired as we expect pairs of sequence to share a similar genomic environment e.g. similar mutation rates. We also compare the aggregate of all ERVs in our study to the aggregate of all repetitive and selfish DNA in our study. This aggregation disassociates paired ERV and flanking sequence, yet an elevated divergence effect is still visible for ERVs. We found the difference between PV and RM+ under aggregation to be 0.0005 substitutions per site i.e. effectively the same as the small median difference between paired autosomal PV and RM+ sequence of 0.001 substitutions per site that we mention above. Nevertheless, all repetitive and selfish DNA discussed so far originated from a location within 40 kb of a full-length ERV by experimental design.

**Fig. 6:**
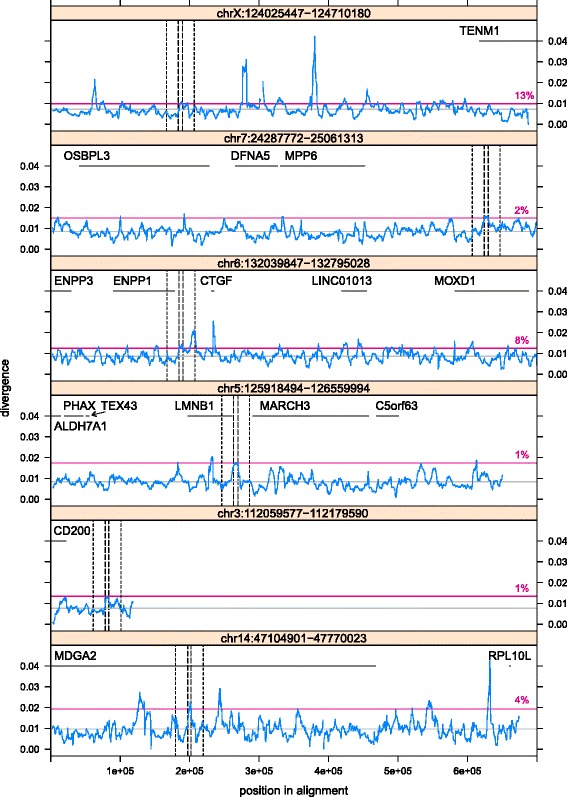
Divergence of six long alignments containing “highly active” HERV-H loci. The divergence of RM+ (selfish DNA) *including* HERV-H sequence (PV) is plotted (blue line) against alignment coordinates using a sliding window of the same length as the HERV-H in each alignment. The grey horizontal line represents the mean divergence of RM+ across the alignment. The magenta horizontal line is a reference line indicating the divergence of the window centred on the HERV-H (i.e. the divergence of PV); the associated percentage gives the percentage of windows for which divergence is at least as great as the divergence of the HERV-H. Inner vertical dashed lines mark a window centred on the HERV-H. Outer vertical dotted lines mark a region of length 40 kb that is centred on the HERV-H. RefSeq gene annotations appear in black

Given the high divergence of HERV-H orthologues, we conducted an additional analysis targeting the six highly active HERV-H orthologues that could be located in long primate alignments. Our motivation was to explore whether ERVs drawn from the fastest diverging group in our study could still be considered to be diverging quickly if we compared them to RM+ regions located at greater distances. This analysis revealed that HERV-H orthologues were local divergence maxima (Fig. 6) and also that an equivalent or greater divergence occurs only when analyzing regions centered on 1–13 % of the loci in these alignments. Furthermore, examining the neighborhood of the ERVs it is clear that they are not located exclusively in regions that are otherwise slowly evolving (plots for ch5 and ch7 reveal nearby sequence that diverges at greater than the alignment mean) but neither are they located exclusively in regions that evolve quickly as a whole (plots for ch14 and chX reveal nearby sequence that diverges at less than the alignment mean). These analyses suggest that our results are not a consequence of ERVs (PV) depressing the divergence of nearby repetitive and selfish flank (RM+). Additionally, as these results indicate that we could find regions that diverged either faster or slower than any particular ERV if we looked far enough away, they support our decision to consider regions that are close to and of a comparable length to ERVs in our other analyses.

Other factors beside selection can influence substitution rates. These include a mutation bias that means that GC nucleotides preferentially decay into AT nucleotides and biased gene conversion. The effect of biased gene conversion may be quite small, but it can be expected to favour the segregation of GC over AT nucleotides. We investigate these two effects below.

Interestingly, for RM+ sequences, we found that divergence was not significantly correlated with GC content for both the CH category (Pearson’s product–moment correlation, r = −0.13, p = 0.07) and for the CH+ category (Pearson’s product–moment correlation, r = −0.01, p = 0.86). We further found that, for PV sequences, divergence was not significantly correlated with GC content for either the CH category (Pearson’s product–moment correlation, r = 0.05, p = 0.48) or for the CH+ category (Pearson’s product–moment correlation, r = −0.07, p = 0.37). This demonstrates that GC content has not driven the divergence of the ERVs or nearby selfish DNA in our dataset (indeed, it is visually clear that different ERV families maintain distinct GC compositions on the timescale of our study as is shown in Figure S1 in Additional file 1). Nevertheless, as we observed that a large fraction of young CH ERVs with larger differences between PV and RM+ divergence were classified as HERV-H (Fig. 2), a family with relatively high GC content, we also performed AIC forward-backward stepwise model selection with the log of the ratio of PV divergence to RM+ divergence as a response variable and age (CH/CH+), ERV family (HERV-H or not HERV-H), and the log ratio of PV to RM+ GC content as explanatory variables. We found that ERV family was the only significant predictor retained by this process, further evidence that the faster evolution of ERVs (PV) compared to their neighbouring selfish DNA (RM+) was not due solely to differences in GC content.

As both mutation bias and gene conversion would act to introduce differences that changed GC content, we also divided all substitutions (EQ+/−) into equilibrating mutations (EQ+) between G or C and A or T and non-equilibrating mutations between G and C or A and T (EQ-) (Table 4). Consistent with the above results, for mutations that were EQ+, we found that PV sequence evolved faster than RM+ sequence that in turn evolved faster than RM- sequence. In contrast, we found that in the EQ- category, RM+ sequence actually diverged slightly more than PV sequence on both the autosome and the X-chromosome. We note that transitions are excluded from the non-equilibrating EQ- category, and that sequences diverge roughly ten-times less when only these substitutions are considered.

As we had observed that censoring CpGs reduced divergence by up to 0.006 substitutions per site we examined the dinucleotide composition of our data (Additional file 1: Table S2 and Figure S2). We found that the dinucleotide composition of PV sequence differed significantly from RM+ and RM- sequence and so we cannot formally rule out the possibility that the greater divergence of PV versus RM+ is due to unidentified context dependent effects.

## Discussion

We have shown that endogenous retroviruses (ERVs) have diverged more at the nucleotide level than other selfish DNA since the chimp-human split. We have further shown that this effect is positively correlated with both the length of an ERV’s LTR and with the percentage of an ERV’s neighbouring DNA that is non-repetitive or non-selfish. The faster evolution of ERVs is especially noticeable for younger members of the HERV-H family, in which case the relative divergence of an ERV when compared to neighbouring selfish DNA correlates well with the level of transcription of the ERV in human stem cells. Our results show a hierarchy of divergence, with ERVs having diverged more than selfish DNA, which in turn has diverged more than non-repetitive or repetitive but non-selfish sequence. We have attempted to rule out mechanistic explanations for our observations and suggest that directional selection is responsible for our results. If the higher divergence of ERVs when compared to other selfish DNA is due to selection then the relative rate of evolution on the autosome compared to the X-chromosome suggests that the mutations that are acted upon are, on average, recessive in nature.

One explanation for selection leading to a faster substitution rate into ERVs than other selfish DNA relates to the cost of an ERV’s mechanism of replication. More than a dozen ERVs in the human genome contain open reading frames [11, 20] but none of the consensus sequences from the ERVs we examined (where present in more than two species) did. This accords with the notion that ERVs are generally fragmented. However, ERVs can have many effects that do not depend on complete coding genes. In general, ERVs can act as promoters or enhancers in opposition to the interests of the host by recruiting transcription factors and interfering with the regulation of nearby host genes [8, 28]. The effect of this kind of disruption can be severe, as is the case for Hodgkin’s lymphoma, which appears to be conditional upon the de-repression of MaLR LTRs [29]. The transcription of ERVs also diverts RNA polymerase from host genes and produces mRNA that may interfere with the preferred regulatory dynamics of the host cell [11]. In some cases such transcripts are known to trigger harmful autoimmune responses such as those that occur in TREX1 deficient mice [30] while in other cases transcripts have been shown to hybridize to produce replication competent (pathogenic) viruses [31, 32]. For fixed ERVs, these kinds of disruption are likely relatively rare or of mild effect, and this is consistent with the observation that in general, the relative divergence of ERVs (as compared with selfish DNA) implies only small selective coefficients. Our observation that ERVs that are surrounded by more selfish DNA diverge more slowly than those surrounded by more non-selfish or non-repetitive DNA is consistent with the idea that the extra mutations we observe in ERVs may be mitigating the transcriptional disruption ERVs cause to nearby host sequence. So, some of the excess divergence we see in ERVs may be due to their remaining capability to recruit transcription machinery and produce transcripts.

There is another reason we might expect selection for substitutions into ERVs, and this relates to an ERV’s repetitive nature, a property shared by all selfish DNA. As repetitive sequences, ERVs can increase the probability of harmful ectopic recombination [9, 33]. The effects of such recombination can be catastrophic to the host, for example, infertility [34, 35]. Using population data, it has been concluded that negative selection acting against full-length polymorphic members of the human specific L1 Ta1 subfamily of LINEs is roughly 2 × 10^−4^ [3]. This is one order of magnitude larger than the largest median selective coefficient we derive using the same effective population size. We do not expect fixed ERVs to cause as much harm via ectopic recombination as LINEs that are removed from the population before fixation, however, we do find that the relative divergence of (whole) ERVs increases with LTR length. Our finding might be due to longer LTRs acting as better promoters, however, it is also consistent with the hypothesis that longer LTRs are more likely to ectopically recombine. This is an idea supported by evidence that purifying selection against TEs in *Drosophila melanogaster* increases with element length [4]. The fact that we found no similar correlation between ERV length and divergence may reflect the fact that the probability of ectopic recombination increases with the number of possible pairings of near-identical elements present in an individual, and therefore roughly with the square of element number. As most ERVs are present only as solo-LTRs, and as each full-length ERV includes two LTRs, the probability of recombination between LTRs is expected to be very much greater than the probability of recombination between other viral regions. Therefore, in short, there is both evidence and reason to believe that ectopic recombination may make some contribution to increasing the rate of divergence between orthologous ERVs.

In this study, we have made comparisons between ERVs (PV) and selfish DNA (RM+). This seemed like a pragmatic way to obtain selection coefficients that characterized the differences between ERVs and sequence that is usually assumed to evolve neutrally. However, it should be noted that our assignment of sequence to one of three categories is crude and suggests that the differences we have reported between ERVs and their surrounding DNA may underestimate the selective forces acting upon ERVs. We have argued that ERVs diverge at faster than neutral rates because they sometimes have an effect on the host, even after fixation. Some of these effects are due to properties shared by most TEs, particularly the potential for ectopic recombination or the disruption of transcription. If ERVs diverge faster than other selfish DNA in part because of properties they share with other TEs, then some portion of TEs should also be expected to diverge at faster than neutral rates. These TEs are assigned to the RM+ category and therefore we compare ERVs to sequence that is, on average, potentially also evolving at faster than neutral rates. For this reason we consider our selection coefficients conservative lower bounds.

The primary goal of this study was to determine whether, on aggregate, ERVs (PV) have had a measurable effect on their hosts. Under our assumptions this could have been seen in one of two ways. First, ERVs could have been conserved relative to neutral (RM+) rates. Second, ERVs could have diverged more quickly than neutral rates. In fact, we observed the second possibility. It is interesting that this is the case but this is not the whole story. We can compare the divergence of ERVs (PV) and selfish DNA (RM+) to non-repetitive or repetitive but non-selfish flank (RM-). Doing so reveals that the distribution of RM+:RM- is shifted to the left of and more peaked than that of PV:RM- (Additional file 1: Figure S3). In other words, relative to non-repetitive or non-selfish DNA, some ERVs diverge more slowly than most other selfish DNA, even though the average ERV is a faster evolver. (The *syncytins* [36, 37] are not part of our dataset but are ERVs that would presumably exhibit such behaviour.) These issues have not been a focus of our study but warrant further investigation because if fixed ERVs have a different distribution of effects to other TEs then they probably have different kinds of effects too. In particular, they may be more often co-opted than other TEs.

Not all of the effects we observed were small. In particular, we observed that the median relative divergence of highly transcribed HERV-H implies a selection coefficient of 5.7 × 10^−5^. This is closer to the selective force acting on a polymorphic LINE and is large enough to be of interest. This is particularly true as we know that highly transcribed HERV-H ERVs are functional components with respect to the regulation of stem cell identity [24]. As we have shown that the relative divergence of HERV-H increases with their transcriptional activity we suggest that the excess substitutions we observe are tuning the transcription levels of these ERVs in stem cells. What is less clear is whether such tuning is associated with adapting pre-existing, necessary and stable host functions [38], or whether it is instead alleviating the cost of transcription as a side effect of the co-option of a subset of HERV-H [11]. For example, it may be that the HERV-Hs that we observe evolving quickly are doing so because they promote functional lncRNAs or chimeric transcripts at a level that needs to be adjusted. Such adjustment might have been necessary due to differences between the biological challenges faced by human, chimpanzee and their common ancestor. On the other hand, it may be that the co-option of some functional HERV-H loci brought with it the unfortunate side effect of the transcription of some different and purely selfish HERV-H loci. These loci would not be at all useful to the host yet could, at an early stage of a host’s lifecycle, introduce any of the previously discussed costs of ERVs. Selection on the host population would be expected to attenuate these costs over time. These two possibilities will in future need to be disentangled, but whatever the reality, we can see that actively transcribed HERV-H has been diverging particularly quickly at the sequence level since the chimp-human split and conclude that our selective coefficient provides a lower bound on the magnitude of the forces acting upon it.

## Conclusions

Endogenous retroviruses (ERVs) have evolved faster than other selfish DNA in human and chimpanzee. The divergence of ERVs relative to neighbouring selfish DNA is positively correlated with the length of the long terminal repeat of an ERV and with the percentage of neighbouring DNA that is non-repetitive or non-selfish. Members of the HERV-H family evolve particularly fast and in a manner that correlates with their level of transcription in human stem cells. Assuming faster evolution is due to directional selection, the typical substitution into an ERV is recessive and a substitution into a highly transcribed HERV-H has a selective coefficient of the order of 10^−4^, which is not small. This suggests that the HERV-H transcriptome has recently evolved under the influence of directional selection. Further work is needed to discover whether HERV-H is the subject of adaptive regulatory change or whether co-opting some proportion of ERVs has opened up the genome to the harmful effects of other unwelcome retrovirally derived guests.

## Methods

### Detecting ERVs

A library of 771 viral *pol* genes were used as probes in a tBLASTn [39] search against five soft-masked primate genomes: human (*Homo sapiens*), chimpanzee (*Pan troglodytes*), gorilla (*Gorilla gorilla gorilla*), orangutan (*Pongo abelii*) and macaque (*Macaca mulatta*). The genomes were obtained from the Ensembl project [40]. The viral probes were selected to represent endogenous and exogenous retroviruses from a broad range of sources and are the same as those used in previous studies [12, 41, 42]. The aim was to identify as many ERVs as possible and a summary of the diversity of probes is available in Additional file 1: Tables S3 and S4. The 15kbp of sequence centred on each of the resulting collection of 19,945 putative *pol* hits was processed using the LTR detection and annotation software LTRharvest and LTRdigest [43]. The original genomic location of the 5′ start and 3′ finish of each LTR was recorded for those regions containing paired LTRs. Locations containing at least one retroviral gene (as detected by LTRdigest) beyond the *pol* identified by tBLASTn were assumed to contain full-length proviruses and were retained for further processing. Our goal was not to identify novel ERVs and confirmation that the location of our ERVs overlap with another study, as well as details of the locations identified by our study, are contained in machine readable form in Additional file 2.

### Detecting orthology between proviruses

Orthologue detection proceeded in two stages. First, the 20kbp surrounding each putative full-length provirus (hereafter 20kbp excerpt) was used as a BLASTn query in a search against every other syntenic 20kbp excerpt from every primate species. Synteny mapping was based on chromosome name and therefore pairings could be made between ERVs on human chromosome 2 and ERVs on chimpanzee chromosomes 2a or 2b. A local BLASTn alignment of at least 7500 nucleotides in length and of at least 95 % identity between two 20kbp excerpts was considered suitable to qualify pairs of 20kbp sequence as potentially orthologous. Second, the aforementioned candidate orthologies were investigated in detail by performing Needleman-Wunch pairwise global alignment using the stretcher program (gap-open penalty 16, gap-extend penalty 4 and matrix EDNAFULL) from the EMBOSS software suite [44]. A sample of over fifty candidate orthologies, picked uniformly at random, were examined by hand. Upon inspection of these pairwise alignments it was determined that choosing a minimum global identity of 85 % and minimum global similarity of 85 % would sufficiently capture our intuition of orthology. That is to say, a lower threshold would run the risk of pairing non orthologous sequence but a higher threshold would unnecessary exclude genuinely orthologous provirus and flank from our study. Alignments of this kind (i.e. alignments indicating orthology) were noted. In the rare event that two or more 20kbp excerpts were orthologous within the same species (a potential paralogy) all homologous 20 kb excerpts across all species were excluded from further analyses. This resulted in the removal of 32 paralogous pairs.

### Annotating aligned provirus and flanking DNA

Once orthology had been determined we switched to using 40kbp excerpts (this did not involve discarding any data). Orthologous 40kbp excerpts were pairwise aligned with the stretcher program using the same settings as mentioned above. Each 40 kb alignment was annotated as follows. We classified each column of our alignment as one of PV, RM+ or RM-. Membership of PV was determined by taking the union of the two contiguous regions identified as an ERV due to running LTRharvest on each of the chimpanzee and human sequences in an alignment. The outermost 25 bp of this union region was excluded from all analyses to take account of uncertainty over the ability of LTRharvest to sharply identify the precise endpoints of 5′ and 3′ LTRs. The remaining flanking columns of each alignment were then classified based on their RepeatMasker annotation. We obtained RepeatMasker annotations for all of our 40kbp excerpts by submitting them to repeatmasker.org using settings “cross_match” and “speed/sensitivity slow”. The category RM+ contained sequence classified as DNA, LINE, Low_complexity, LTR, RC, Retroposon, Satellite, Simple_repeat, SINE or Unknown; the category RM- contained unmasked sequence or sequence classified as RNA, rRNA, scRNA, snRNA, srpRNA or tRNA. All dinucleotide pairs in an alignment were annotated as CpG sites if they were zero or one mutation away from CG:CG or GC:GC, i.e. exhibited a potentially mutated cytosine or guanine, or if they were of the form TG:CA or CA:TG, i.e. exhibited a potential common double transition at both cytosine and guanine.

### Alignment quality

When performing distance calculations we were concerned with ensuring that, as far as possible, differences between sequences did not result from regions of bad alignment. To mitigate this possibility we excluded gapped and low complexity regions from our final analysis using a program (available on request) that implemented the following heuristic method. Alignments were broken into blocks separated by gaps or low complexity regions of eight or more consecutive columns in length. Low complexity sequence was defined as that masked by the dustmasker program of the BLAST suite [39]. The edges of blocks of ungapped and unmasked sequence were examined six nucleotides at a time. If these six nucleotide regions contained any mismatched bases the appropriate block had the six nucleotide region removed. This process was repeated until blocks started and finished with regions containing six identical nucleotides or were removed entirely. Only blocks of at least 20 nucleotides in length were used in our analyses.

### Calculating distances

All distances were calculated using PAML 4.8 [45]. For per-alignment comparisons the K80 method was used. For aggregate comparisons both the K80 and the GTR model were applied, though we found the two methods produced identical distances beyond the precision reported in our study. The overall number of patterns used to calculate distances appear in Table 3 and Additional file 1: Table S1.

### Calculating selection coefficients

Assuming substitutions into RM+ are neutral then a measure of the rate of substitution in the RM+ flank is also a measure of the neutral mutation rate *u*. We write the elevated substitution rate into ERV DNA that we obtain from measures of divergence of PV+ as γ. It is well known that the ratio λ = γ / *u* is directly related to the selection coefficient *s* acting on substitutions. Therefore, under the assumption of weak selection, a Wright-Fisher model of drift and semi-dominant mutations (*h* = 1/2) we have: λ = 2 *N*(1 – exp(−*s*)) / (1 – exp(−2*Ns*)). As the diffusion equation from which the previous equation is derived assumes a small *s*, it is common and numerically convenient to use the approximation λ = 2*Ns* / (1 – exp(−2*Ns*)) [46]. We take effective population size *N* = *N*_*e*_ to be 10,000 in our calculations [3].

### Calculating dominance

By calculating the relative divergence of autosomal and X-linked ERVs it is possible to make statements about dominance [47]. Denote the rate of substitution of mutations on the autosome as *K*_*A*_ = 2*Nv*_*A*_*u*_*A*_, where 2 *N* is the number of copies of the autosome in a population, *v*_*A*_ is the probability of fixation of a beneficial mutation, and *u*_*A*_ is the mutation rate. For the X chromosome the analogous expression is *K*_*X*_ = 3/2 *Nv*_*X*_*u*_*X*_, where we allow substitutions on the X chromosome to derive from a process with its own mutation rate and probability of fixation.

Alignments of orthologous sequence provide chimp-human divergence values *K*_*A*_*t* (autosomal PV), *u*_*A*_*t* (autosomal RM+), *K*_*X*_*t* (X-linked PV) and *u*_*X*_*t* (X-linked RM+), where *t* is the evolutionary time for which the chimpanzee and human have been separated. Let ratios of divergence be denoted by *A* and *X* so that *A* = *K*_*A*_ / *u*_*A*_ and *X* = *K*_*X*_ / *u*_*X*_. Using aggregated data we find that *X* > *A* (see Results section).

Assuming weak directional selection, and the population genetic framework in Additional file 1: Table S5, which allows separate selective coefficients *s*_*m*_ in males and *s*_*f*_ in females, the probabilities of fixation *v*_*A*_ and *v*_*X*_ are well approximated by 1/2 *h*(*s*_*f*_ + *s*_*m*_) and 1/3(2*hs*_*f*_ + *s*_*m*_) respectively [48]. These weak selection approximations allow one to make statements about dominance and sexually antagonistic selection. Based on our divergence data we are interested in cases when 2 *h*(*s*_*f*_ + *s*_*m*_) < 2*hs*_*f*_ + *s*_*m*_. For positive *s*_*m*_, this occurs when (dominance) *h* < 1/2.

### Transcription data

We paired genomic coordinates located in the supplementary material of [24] with the genomic coordinates of our 40kbp excerpts from human. Each HERV-H locus in [24] was paired with its nearest syntenic 40kbp excerpt from human if the distance between the centroids of the two sets of coordinates (theirs and ours) was less than 2500 bp. This resulted in the association of 63 of the 64 of the previously identified HERV-H ERVs in our dataset with 63 sets of transcription data. No association between transcription data and ERVs from any other family was made. The nominal transcription levels “highly active”, “moderately active” and “inactive” are the same as those referred to in the main text and figures of [24] and were read directly from the supplementary data. The continuous levels we discuss were obtained by taking the mean of the expression levels across all stem cell measurements in the supplementary data [24].

### Long distance analysis

To examine the divergence of regions greater than 40 kb in length we searched the six-way EPO multiple alignments available from the Ensembl project for regions that contained the coordinates of the 12 “highly active” HERV-H orthologues in our study. Alignments for six of the 12 orthologues could be identified. We removed sequence that was gapped in both chimpanzee and human. We then annotated the chimpanzee and human sequence in the same way as our 40 kb alignments (described above). For each of the six alignments we computed the divergence of sites classified as RM+ or PV using a sliding window. For any particular alignment we used a natural window size of the same length as the HERV-H region the alignment contained.

## Additional files

Additional file 1:
**Contains supplementary figures and tables.** **Table S1.** Site patterns observed across non CpG censored alignments. Patterns were observed at sites classified as one of: ERV (PV); selfish DNA (RM+); or non-repetitive or repetitive but non-selfish (RM-). **Table S2.** Dinucleotide pattern counts. Patterns were observed at sites classified as one of: ERV (PV); selfish DNA (RM+); or non-repetitive or repetitive but non-selfish (RM-). Pattern classification was performed on both CpG censored and non CpG censored data. **Table S3.** Source organisms for *pol* probes used in this study grouped by ERV class of virus. **Table S4.** Viral diversity of *pol* probes used in this study. **Table S5.** The model of fitness effects of mutations into ERVs (PV) used in this study. **Figure S1.** GC content of PV region by ERV family. ERV family was assigned using the best matching viral *pol* probe (see Detecting ERVs in Methods and Additional file 2). **Figure S2.** Dinucleotide frequencies grouped by sequence classification and transition count: no transition (0); single transition (1); double transition (2). **Figure S3.** Distribution of divergence of ERVs (PV) and other selfish DNA (RM+) versus paired non-repetitive or non-selfish flank (RM-). Additional file 2:
**Contains information on the genomic location, family and orthology of the ERVs used in this study.**
