# Supplementary material for: Orthologous endogenous retroviruses exhibit directional selection since the chimp-human split
Source: Retrovirology. 2015 Jun 20;12:52. doi: 10.1186/s12977-015-0172-6 (PMC4477479; doi:10.1186/s12977-015-0172-6)

Supplementary data

Table S1. Site patterns observed across non CpG censored alignments. Patterns were observed at sites classified as one of: ERV (PV); selfish DNA (RM+); or non-repetitive or repetitive but non-selfish (RM-).

chimp:human	autosomal			X-linked		
	PV	RM+	RM-	PV	RM+	RM-
A:A	591392	1603551	1145366	26509	83350	43123
A:T	888	2496	1390	38	105	45
A:G	6651	14093	7336	242	532	225
A:C	1301	3104	1860	53	127	55
A:?	0	0	0	0	0	0
T:A	984	2572	1456	28	90	41
T:T	597941	1609580	1151417	26035	87639	43695
T:G	1396	3302	1787	58	125	68
T:C	6507	13999	7213	241	558	247
T:?	0	0	0	0	0	0
G:A	6019	13776	7188	241	585	214
G:T	1364	3528	2002	47	131	56
G:G	476441	1189167	734803	23851	59232	25988
G:C	1481	3531	1942	57	146	73
G:?	0	0	0	0	0	0
C:A	1449	3442	1914	38	117	58
C:T	6153	13605	7195	216	515	249
C:G	1420	3558	1858	54	119	72
C:C	486320	1182717	729658	19527	58921	26391
C:?	0	0	0	0	0	0
?:A	5	0	2	0	0	0
?:T	0	1	2	0	1	0
?:G	1	0	1	0	1	0
?:C	1	4	0	0	0	0
?:?	0	0	0	0	0	0
total	2187714	5666026	3804390	97235	292294	140600

Table S2. Dinucleotide pattern counts. Patterns were observed at sites classified as one of: ERV (PV); selfish DNA (RM+); or non-repetitive or repetitive but non-selfish (RM-). Pattern classification was performed on both CpG censored and non CpG censored data.

	uncensored			censored		
pattern	PV	RM+	RM-	PV	RM+	RM-
AA-GG|TT-CC|GG-AA|CC-TT	67	127	73	36	85	58
AC-AG|AG-AC|GT-CT|CT-GT	1821	4630	2649	1441	3921	2245
CG-GA|TC-CG|GA-CG|CG-TC	24	39	17	20	33	17
GC-AA|GC-TT|AA-GC|TT-GC	32	95	38	21	70	32
AA-AT|TT-AT|AT-TT|AT-AA	1020	2941	1698	1020	2941	1698
GA-AA|TC-TT|AA-GA|TT-TC	4988	11606	6371	2864	7494	4695
CA-CC|TG-GG|CC-CA|GG-TG	1678	3807	1895	1410	3172	1568
AA-CC|TT-GG|CC-AA|GG-TT	16	59	22	13	47	21
TG-TT|AA-CA|TT-TG|CA-AA	1684	4456	2627	1684	4456	2627
CT-CA|TG-AG|CA-CT|AG-TG	1098	2773	1344	1098	2773	1344
CG-AA|CG-TT|AA-CG|TT-CG	28	40	19	28	40	19
AG-TA|CT-TA|TA-CT|TA-AG	13	48	22	13	48	22
GT-CG|CG-AC|AC-CG|CG-GT	7	21	12	6	16	10
GA-CT|TC-AG|AG-TC|CT-GA	4	28	10	4	25	9
TT-TT|AA-AA	379597	1032906	788505	379597	1032906	788505
TT-GT|GT-TT|AC-AA|AA-AC	1513	3743	2321	1267	3230	1992
AC-TT|AA-GT|TT-AC|GT-AA	19	68	25	18	48	20
AA-AG|CT-TT|AG-AA|TT-CT	4967	12324	7438	4967	12324	7438
CC-TC|GA-GG|GG-GA|TC-CC	4251	9014	4818	3995	8651	4635
AG-GT|CT-AC|AC-CT|GT-AG	25	64	34	21	38	24
CG-GC|GC-CG	4	8	3	2	6	1
TT-AG|CT-AA|AG-TT|AA-CT	10	39	14	10	39	14
CT-CC|GG-AG|AG-GG|CC-CT	7385	13982	6660	3783	8107	4422
AG-AG|CT-CT	332695	832303	535497	332695	832303	535497
AG-GC|GC-CT|CT-GC|GC-AG	22	67	28	10	40	22
CA-AG|CT-TG|AG-CA|TG-CT	102	227	91	102	227	91
CT-GG|CC-AG|AG-CC|GG-CT	12	38	18	8	34	14
TA-GC|GC-TA	2	9	5	2	8	5
**AG-CG|CG-CT|CT-CG|CG-AG**	896	1977	1128	0	0	0
GC-GC	104712	260685	147021	93799	234971	133678
TA-CG|CG-TA	45	107	37	45	107	37
GA-GA|TC-TC	267882	700469	459056	254279	672557	444606
AT-TA|TA-AT	4	16	11	4	16	11
AT-CT|AT-AG|CT-AT|AG-AT	1462	3650	2172	1462	3650	2172
AG-CT|CT-AG	12	17	12	12	17	12
AC-GG|CC-GT|GT-CC|GG-AC	30	72	16	17	38	8
TC-GA|GA-TC	3	7	9	3	6	5

AC-AC|GT-GT	221387	594562	381313	209715	568402	364188
AT-GC|GC-AT	48	146	57	17	62	30
AT-GA|AT-TC|TC-AT|GA-AT	20	67	27	14	53	20
AC-CC|GG-GT|GT-GG|CC-AC	1381	3078	1529	1320	2967	1456
GA-GC|TC-GC|GC-GA|GC-TC	1184	2790	1404	922	2255	1111
TA-TG|TA-CA|TG-TA|CA-TA	4684	12823	7752	4684	12823	7752
CC-GG|GG-CC	8	17	3	5	13	3
AC-CA|GT-TG|TG-GT|CA-AC	11	41	20	11	36	19
GC-GG|GC-CC|CC-GC|GG-GC	1255	2883	1456	959	2339	1178
GG-TC|GA-CC|CC-GA|TC-GG	6	24	11	4	20	11
CC-CC|GG-GG	291451	631781	357328	274771	601314	340033
AT-CG|CG-AT	2	4	2	2	4	2
**CG-TG|CG-CA|CA-CG|TG-CG**	10365	19695	8493	0	0	0
CA-CA|TG-TG	316037	876553	540776	316037	876553	540776
CC-AT|AT-GG|GG-AT|AT-CC	30	63	33	15	44	22
AC-GT|GT-AC	61	137	56	17	53	31
TG-AT|CA-AT|AT-CA|AT-TG	7	24	12	7	24	12
GA-CA|CA-GA|TC-TG|TG-TC	1642	4399	2452	1392	3869	2199
AG-GA|CT-TC|GA-AG|TC-CT	79	156	76	43	91	56
GC-TG|GC-CA|CA-GC|TG-GC	5	20	10	4	17	8
TA-CC|CC-TA|GG-TA|TA-GG	14	45	23	12	38	22
AT-AT	143864	431368	319021	143864	431368	319021
CA-GG|TG-CC|GG-CA|CC-TG	98	219	62	82	194	60
GA-AC|GT-TC|AC-GA|TC-GT	16	59	20	11	32	12
TG-GA|GA-TG|TC-CA|CA-TC	19	62	20	18	54	19
**CG-CG**	20819	44375	26458	0	0	0
TT-AA|AA-TT	5	22	7	5	22	7
**TG-CA|CA-TG**	718	1179	377	0	0	0
TA-TA	130756	361441	280640	130756	361441	280640
GT-GC|GC-AC|AC-GC|GC-GT	5374	11537	5170	2663	6296	3356
TC-TA|GA-TA|TA-TC|TA-GA	1178	3012	1913	1024	2691	1711
AA-TC|TT-GA|GA-TT|TC-AA	13	27	23	12	25	23
TA-AA|AA-TA|TT-TA|TA-TT	887	2376	1617	887	2376	1617
TT-CA|CA-TT|TG-AA|AA-TG	21	54	17	21	54	17
GT-AT|AC-AT|AT-GT|AT-AC	7953	18962	10935	4667	12300	7413
TA-GT|TA-AC|AC-TA|GT-TA	6	17	11	6	17	11
GA-GT|GT-GA|TC-AC|AC-TC	727	1915	976	694	1838	938
**CG-CC|GG-CG|CG-GG|CC-CG**	1061	2105	1079	0	0	0
TG-AC|AC-TG|CA-GT|GT-CA	10	34	11	10	31	8

Table S3. Source organisms for *pol* probes used in this study grouped by ERV class of virus.

	Class I	Class II	Class III	NA
Anseriformes	1	5	0	0
Anura	9	0	1	0
Apterygiformes	0	4	0	0
Artiodactyla	10	7	0	0
Caecilia	2	0	2	0
Carnivora	30	4	0	0
Casuariformes	0	2	0	0
Caudata	4	0	0	0
Cetacea	3	1	0	0
Chiroptera	27	0	0	0
Chondricthyes	1	0	0	0
Columbiformes	0	1	0	0
Crocodilia	1	0	0	0
Cypriniformes	1	0	0	0
Didelphimorphia	2	0	1	0
Diprotodontia	0	1	0	0
Falconiformes	1	6	0	0
Galliformes	0	10	0	0
Gaviiformes	0	2	0	0
Gruiformes	0	2	0	0
Insectivora	1	1	0	0
Lagomorpha	1	2	0	0
Marsupiala	2	0	0	0
Marsupialia	2	2	0	0
Monotremata	3	2	0	0
Insecta	0	0	0	2
Fungi	0	0	0	2
Passeriformes	1	11	0	0
Perciformes	1	0	1	0
Perissodactyla	1	1	0	0
Phoenicopteriformes	0	1	0	0
Piciformes	0	3	0	0
Pinnipedia	1	0	0	0

Primates	198	51	54	0
Rheiformes	0	2	0	0
Rodentia	249	16	0	0
Scandentia	1	0	0	0
Sphenisciformes	0	1	0	0
Sphenodontia	1	0	1	0
Squamata	4	2	0	0
Strigiformes	0	2	0	0
Struthioniformes	0	1	0	0
Teleostii	1	0	0	0
Testudines	2	0	0	0
Tinamiformes	0	1	1	0
Xenarthra	1	0	0	0

Table S4. Viral diversity of *pol* probes used in this study.

	endogenous	exogenous	exogenous/endogenous
A-type	10	0	0
Alpharetrovirus	2	1	0
Avian-IIA	7	0	0
Avian-IIB	8	0	0
Betaretrovirus	14	3	2
Deltaretrovirus	0	2	0
Dev	3	0	0
Epsilonretrovirus	0	2	0
ERV-9	7	0	0
HERV-AC018462	4	0	0
HERV-AC096774	1	0	0
HERV-ADP	8	0	0
HERV-E	8	0	0
HERV-F	3	0	0
HERV-F type_b	7	0	0
HERV-F type_c	2	0	0
HERV-FRD	2	0	0
HERV-H	57	0	0
HERV-I	14	0	0
HERV-K(HML2)	14	0	0
HERV-K(HML5)	14	0	0
HERV-K(HML6)	9	0	0
HERV-K(HML9)	3	0	0
HERV-L	44	0	0
HERV-L type_b	7	0	0
HERV-P	5	0	0
HERV-R	7	0	0
HERV-R type_b	3	0	0
HERV-R type_c	3	0	0
HERV-S	5	0	0
HERV-T	13	0	0
HERV-U3	1	0	0
HERV-W	16	0	0

HERV-XA	4	0	0
HERV-Z69907	5	0	0
Lentivirus	0	4	0
LPDV-group	3	1	0
LTR-retrotransposons	4	0	0
RRHERV-I	9	0	0
SpeV	1	0	0
spumavirus	0	4	0
Unclassified	416	5	4

Table S5. The model of fitness effects of mutations into ERVs (PV) used in this study.

			male			female		
linkage	A	genotype	A_1A_1	A_1A_2	A_2A_2	A_1A_1	A_1A_2	A_2A_2
		fitness	1	$1 + h\ s_m$	$1 + s_m$	1	$1 + h\ s_f$	$1 + s_f$
	X	genotype	A_1		A_2	A_1A_1	A_1A_2	A_2A_2
		fitness	1		$1 + s_m$	1	$1 + h\ s_f$	$1 + s_f$

Figure S1. GC content of PV region by ERV kind. ERV kind was assigned using the best matching viral *pol* probe (see Detecting ERVs in Methods and Additional File 2).

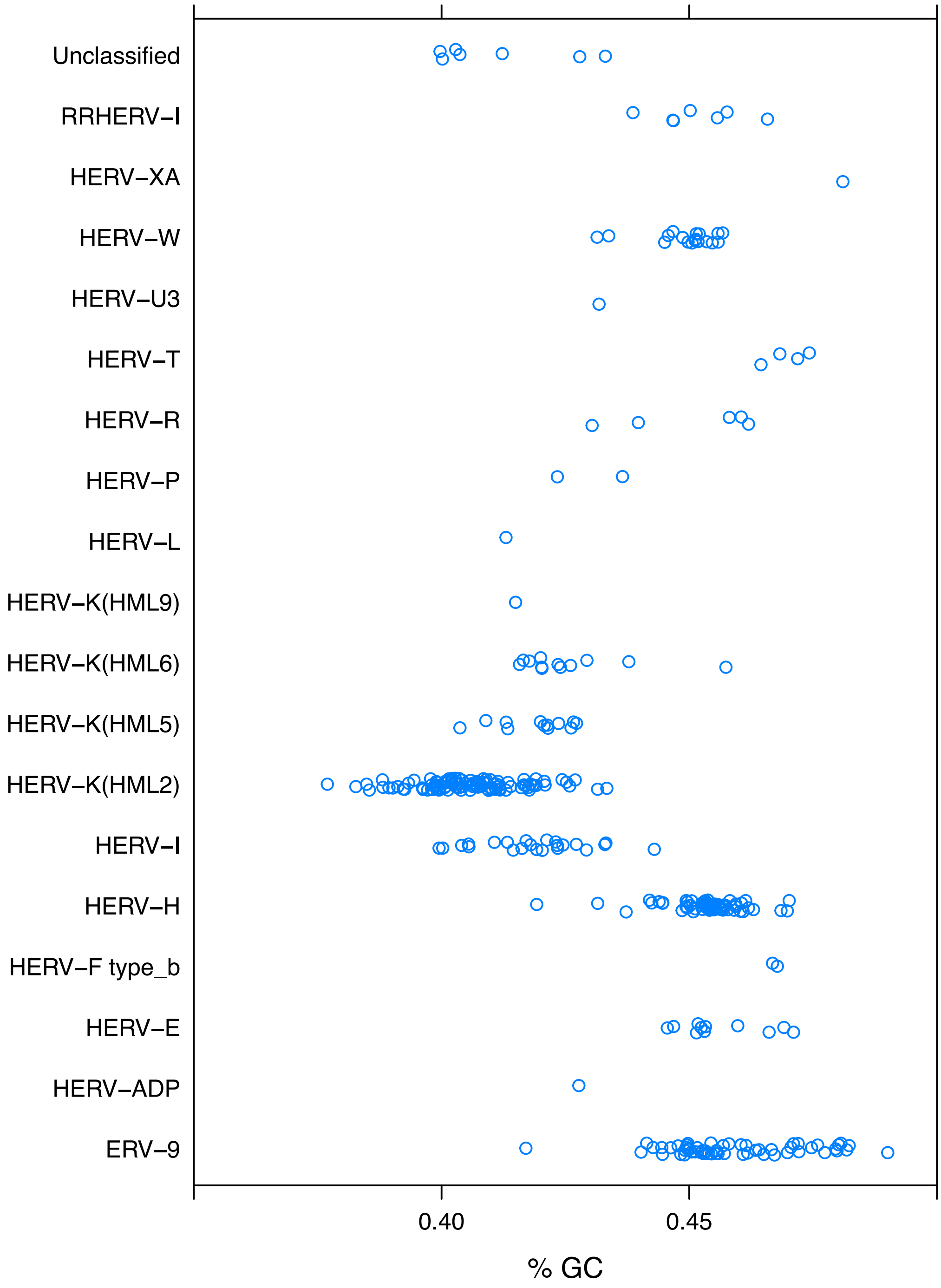

Figure S2. Dinucleotide frequencies grouped by sequence classification and transition count: no transition (0); single transition (1); double transition (2).

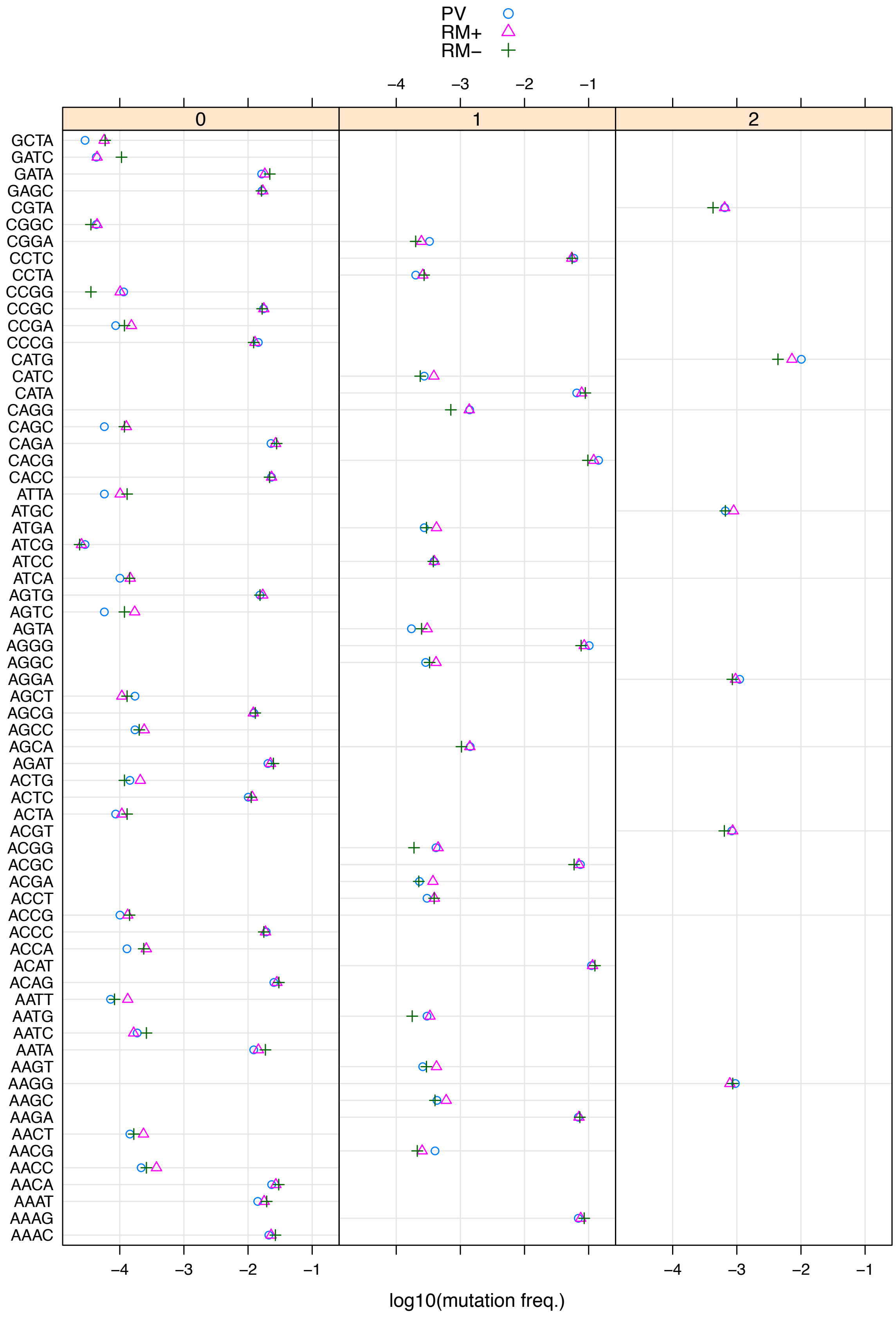

Figure S3. Distribution of divergence of ERVs (PV) and other selfish DNA (RM+) versus paired non-repetitive or non-selfish flank (RM-).

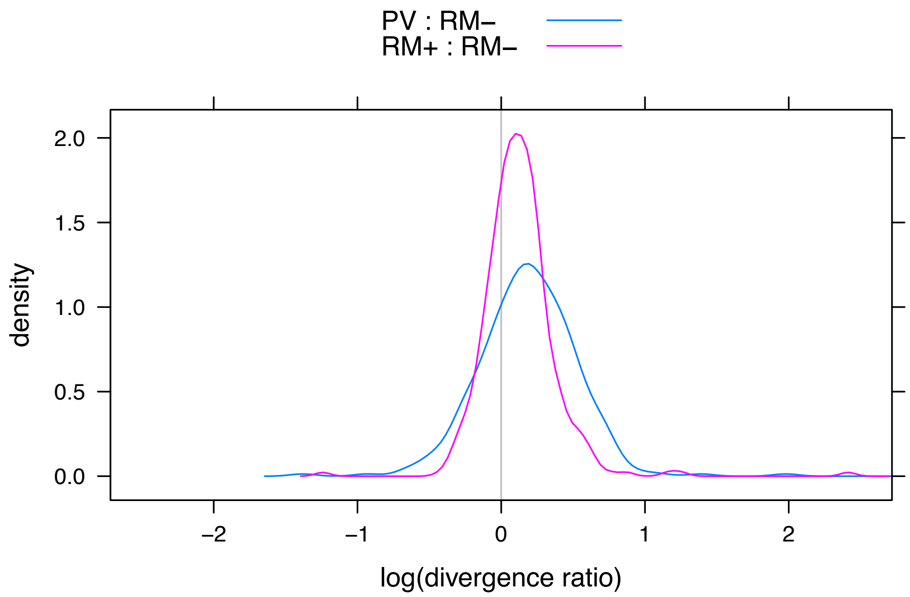

Supplement: Additional file 1: — Contains supplementary figures and tables. Table S1. Site patterns observed across non CpG censored alignments. Patterns were observed at sites classified as one of: ERV (PV); selfish DNA (RM+); or non-repetitive or repetitive but non-selfish (RM-). Table S2. Dinucleotide pattern counts. Patterns were observed at sites classified as one of: ERV (PV); selfish DNA (RM+); or non-repetitive or repetitive but non-selfish (RM-). Pattern classification was performed on both CpG censored and non CpG censored data. Table S3. Source organisms for pol probes used in this study grouped by ERV class of virus. Table S4. Viral diversity of pol probes used in this study. Table S5. The model of fitness effects of mutations into ERVs (PV) used in this study. Figure S1. GC content of PV region by ERV family. ERV family was assigned using the best matching viral pol probe (see Detecting ERVs in Methods and Additional file 2). Figure S2. Dinucleotide frequencies grouped by sequence classification and transition count: no transition (0); single transition (1); double transition (2). Figure S3. Distribution of divergence of ERVs (PV) and other selfish DNA (RM+) versus paired non-repetitive or non-selfish flank (RM-). [file 12977_2015_172_MOESM1_ESM.pdf]
